# Role of diagnostic testing in reducing unnecessary antibiotic use for upper respiratory tract infections in Chinese primary healthcare: a mixed-methods study

**DOI:** 10.1186/s12875-026-03398-z

**Published:** 2026-05-28

**Authors:** Chengzhou Tang, Yushan Zhang, Muhtar Kadirhaz, Sen Xu, Yunshu Lu, Iltaf Hussain, Wei Zhao, Yi Dong, Jiaxu Lin, Runfang Mu, Yang Zhao, Mingwang Shen, Yu Fang, Jie Chang

**Affiliations:** 1 Department of Pharmacy Administration, School of Pharmacy, Xi’an Jiaotong University, Xi’an, China; 2 Center for Drug Safety and Policy Research, Xi’an Jiaotong University, Xi’an, China; 3 Shaanxi Center for Health Reform and Development Research, Xi’an, China; 4 Research Institute for Drug Safety and Monitoring, Institute of Pharmaceutical Science and Technology, western China Science and Technology Innovation Harbor, Xi’an, China; 5 Programme in Health Services Research & Population Health, Duke-NUS Medical School, National University of Singapore, Singapore, Singapore; 6 Department of Epidemiology and Biostatistics, School of Public Health, Joint Research Center for Infectious Diseases, School of Public Health, Xi’an Jiaotong University, Xi’an Jiaotong University Health Science Center, Xi’an, China; 7 Department of Pharmacy Administration and Clinical Pharmacy, School of Pharmacy, Xi’an Jiaotong University, 76 Yanta West Road, Xi’an, Shaanxi Province China

**Keywords:** Primary healthcare, Diagnostic tools, Antibiotic use, Upper respiratory tract infections, Antimicrobial resistance

## Abstract

**Backgrounds:**

Antimicrobial resistance (AMR) is a growing global concern, driven by inappropriate antibiotic use, particularly in primary healthcare(PHC). The treatment of upper respiratory tract infections (URTIs), which are mostly of viral etiology, is a major source of such misuse. Limited access to diagnostic tools may contribute to unnecessary prescribing. This study examines whether the availability of diagnostic tools reduces unnecessary antibiotic use for URTIs in primary healthcare settings of China, with particular attention to facility-level availability of C-reactive protein (CRP) testing.

**Methods:**

A mixed-methods approach was employed, integrating a cross-sectional survey of general practitioners (GPs) with an analysis of their prescription records and in-depth interviews with a subset of GPs. Quantitative analysis involved descriptive statistics and multilevel logistic regression to assess whether facility-level availability of CRP testing was associated with antibiotic prescribing for URTIs, whereas qualitative interviews explored the contextual influences on clinical decision-making.

**Results:**

A total of 108 general practitioners from 30 PHC facilities filled out the questionnaires. Records of 12,489 prescriptions for URTI issued by these physicians were retrieved, of which 7,706 contained at least one antibiotic, with a URTI antibiotic prescribing rate of 61.7%. Among the 30 PHC facilities, 11 (36.7%) were equipped with CRP testing. Multilevel logistic regression models showed that CRP-test availability was associated with lower odds of antibiotic prescribing for URTIs (OR = 0.74; 95%CI:0.56–0.98; *p* = 0.033). Interviews with GPs revealed that limited access to diagnostic tools increased uncertainty in clinical decision-making, whereas access to such tools supported more evidence-informed prescribing and improved communication with patients.

**Conclusions:**

C-reactive protein testing and other diagnostic tools play an important role in enabling rational antibiotic prescribing in PHC facilities. Strengthening diagnostic capacity and ensuring ongoing professional training for GPs represent essential strategies to curb inappropriate antibiotic use, mitigating the progression of antimicrobial resistance.

**Supplementary Information:**

The online version contains supplementary material available at 10.1186/s12875-026-03398-z.

## Introduction

Antimicrobial resistance (AMR) is a major global public health issue associated with 7 million deaths annually. If not adequately addressed, the projected incidence of deaths could surpass 10 million by the year 2050, exceeding even cancer in terms of death rates [1–3]. In China, it has been estimated that AMR contributes to an alarming toll of over 1.3 million deaths annually [4]. China stands as the primary global producer and consumer of antibiotics and presents considerable challenges related to the misuse of antibiotics. According to a systematic review, antibiotics were prescribed in 50.3% of outpatient encounters in China, and prescribing rates in some rural areas were reported to be as high as 70% [5]. Upper respiratory tract infections (URTIs) are common clinical conditions primarily caused by viruses such as influenza and rhinovirus, sometimes followed by bacterial infections [6]. However, research indicates a substantial prevalence of antibiotic over-prescription in China, with approximately 83.7% of outpatients presenting with URTIs receiving antibiotic treatment [7]. This practice yields negligible therapeutic benefits and accelerates the development of AMR.

Diagnostic uncertainty and fear of complications often lead to inappropriate antibiotic prescriptions [8, 9]. Proper diagnostic testing can reduce uncertainty and serve as an antibiotic stewardship strategy [10]. Inadequate diagnostic capacity in many low- and middle-income countries has resulted in excessive reliance on empirical therapy, contributing to pervasive antibiotic use [11]. In recent years, certain biomarkers, such as C-reactive protein (CRP), have increasingly been utilized to inform antibiotic prescribing practices for URTIs [12–15]. CRP is a nonspecific inflammatory marker that may rise in both viral and bacterial infections, but bacterial infections are often associated with higher CRP levels. In primary care, CRP testing is therefore useful not as a standalone diagnostic test, but as a tool to reduce diagnostic uncertainty and support antibiotic prescribing decisions in respiratory tract infections [13, 16, 17]. Previously, it has been reported that the implementation of CRP testing can significantly mitigate the inappropriate utilization of antibiotics by enhancing diagnostic accuracy [16, 18–22]. A study reported from China stated that limited diagnostic knowledge was the primary driver of unnecessary antibiotic prescriptions [9].

Although numerous studies have examined factors influencing antibiotic prescribing, evidence on how the availability of diagnostic tools shapes clinical decision-making in primary healthcare in China remains limited and inconclusive. Therefore, this study aimed to examine the association between the availability of diagnostic tools for URTIs in healthcare facilities in Shaanxi, China. Quantitatively, we assessed whether access to diagnostic tools, particularly CRP testing, was associated with antibiotic prescribing; qualitatively, we explored how diagnostic availability influenced physicians’ prescribing decisions in routine practice.

## Methods

### Study design

A convergent parallel mixed-methods research design was used. The convergent nature of this hybrid approach ensures that quantitative and qualitative data are collected simultaneously, analyzed independently, and the results of the study are integrated for interpretation [23].

**Fig. 1:**
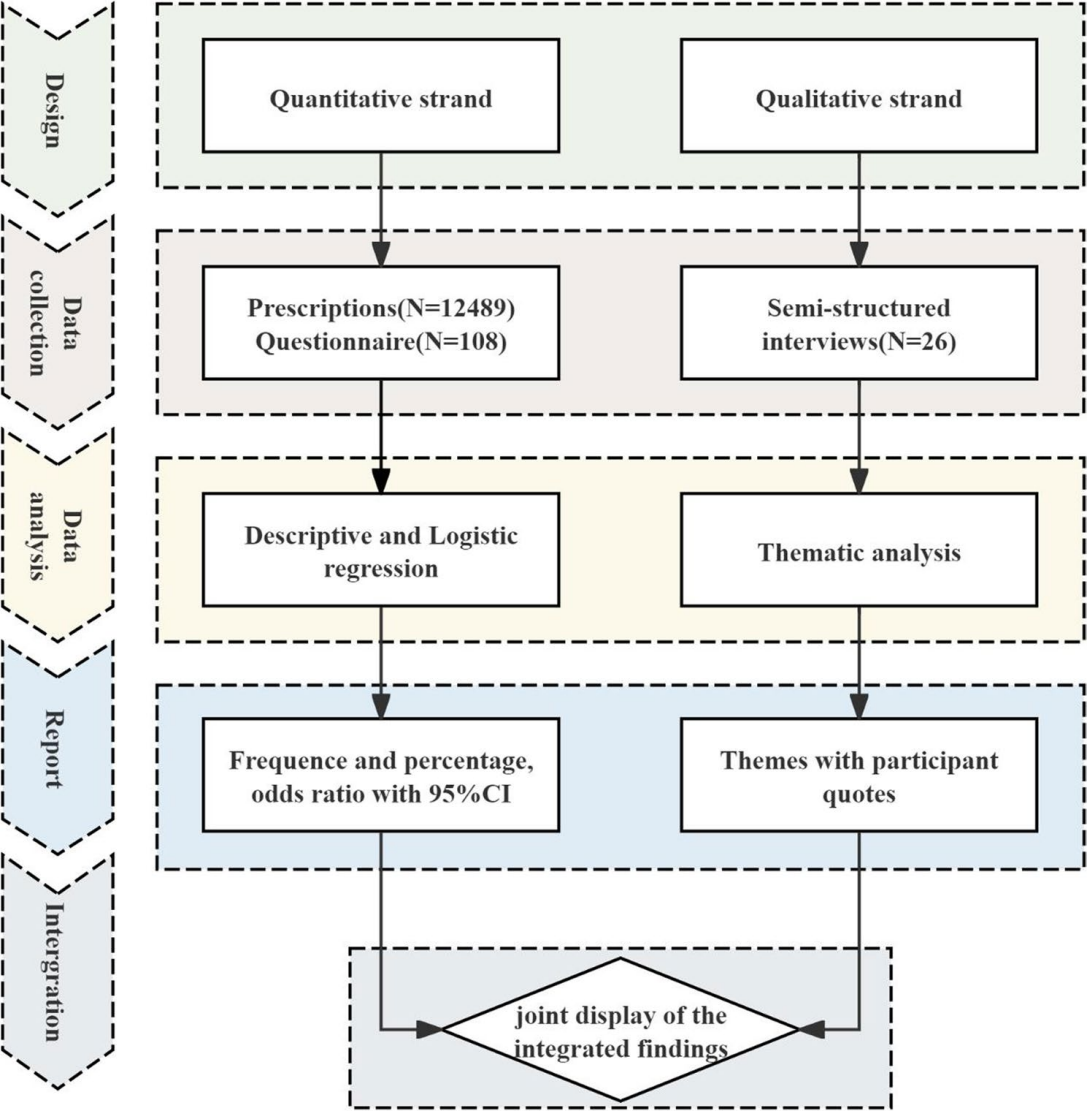
Convergent mixed-methods design procedure flowchart

Data on potential determinants of antibiotic use and physicians’ attitudes toward diagnostic tools and empirical antibiotic treatment for URTIs were obtained from prescription records and questionnaire surveys. The qualitative data derived from semi-structured interviews explored physicians’ perspectives on the utilization of diagnostic tools for URTIs, their rationale for empirical antibiotic prescribing, and the perceived relationship between these practices. Both quantitative and qualitative data sets were accorded equal priority, and integration occurred during the interpretation phase to achieve a more comprehensive understanding of how diagnostic tool availability shapes rational antibiotic use for URTIs (Fig. 1).

### Study setting and sampling

This study was conducted in Shaanxi Province, located in western China, which has a resident population of 33.55 million and spans 205,600 square kilometres. In 2020, Shaanxi Province’s annual gross domestic product (GDP) was 2618.18 billion yuan, ranking 14th among 31 provinces across the country [24]. We purposely selected three out of ten prefecture-level regions in Shaanxi Province based on their geographic location and economic level. These regions included Xi’an from the central part of Shaanxi, with the highest GDP ranking in the province; Yan’an, located in northern Shaanxi, with a GDP ranking of 6th; and Shang’luo from southern Shaanxi, with a GDP ranking of 9th among the ten prefecture-level cities in the province. We then used convenience sampling to recruit PHC facilities in each selected prefecture-level region. Ultimately, 30 PHC facilities participated in our study.

Physicians were recruited from the 30 participating PHC facilities. First checked whether physicians working in these facilities had the authority to prescribe antibiotics. A total of 119 physicians were initially screened. Physicians were eligible if they had antibiotic-prescribing authority and had issued at least 15 outpatient prescriptions per month during the study period. Clinicians without prescribing authority or with fewer than 15 prescriptions per month between 1 January 2020 and 31 December 2020 were excluded. This resulted in a final sample of 108 GPs. Members of the research team visited the participating PHC facilities and distributed the questionnaires to physicians face-to-face. For the qualitative component, a nested sampling approach was used to select GPs for in-depth interviews from the participating PHC facilities, with priority given to doctors who were on duty on the interview day. One GP from each of the 30 participating facilities was invited to take part, and interviews continued until theoretical saturation was reached. In total, 26 GPs participated in the interviews, including 8 from Xi’an, 9 from Shang’luo, and 9 from Yan’an. No financial compensation was provided.

Prescription data were then extracted from the hospital information system for the included physicians. We retrieved outpatient electronic prescriptions issued between 1 January 2020 and 31 December 2020 for visits diagnosed as acute upper respiratory tract infection. Prescription records included the generic names of prescribed medicines, the anonymized identifier of the prescribing physician, and patients’ basic information, including age, gender, diagnosis, and payment method. Multiple prescriptions for the same patient on the same day in the same facility were treated as a single visit. In total, 12,489 eligible URTI prescriptions were included in the analysis. The details of prescription extraction principles are provided in Additional file 1. A.

### Quantitative study

#### Questionnaire design

The questionnaire comprises four sections: socio-demographic and facilities characteristics, perception of diagnostic tool availability for URTIs, attitudes towards the use of diagnostic tools for URTIs, and attitudes to empirical antibiotic treatment of patients with episodes of illness. Perception of diagnostic tool shortages for URTIs was assessed using three questionnaire items rated on a 7-point scale from 1 (“never”) to 7 (“always”). For analysis, responses of 2–7 were interpreted as indicating that the issue occurred at least occasionally. Attitudes towards the clinical use of diagnostic tools were assessed using 10 Likert-type items. Items 1–5 were positively worded and scored from 1 (“strongly disagree”) to 5 (“strongly agree”), whereas items 6–10 were negatively worded and scored from 1 (“strongly agree”) to 5 (“strongly disagree”), so that higher values consistently reflected more positive attitudes. Total scores ranged from 10 to 50, with higher scores indicating more positive attitudes. The full wording of all questionnaire items and response options is provided in Additional file 1. B.

#### Prescription data

Additionally, prescriptions related to URTIs by GPs between 1 January 2020 and 31 December 2020 were also collected. Prescription details included the generic name of the medicines prescribed, the unique identity number of the prescribing physician (anonymized and used only for identification), and patients’ basic information (age, gender, diagnosis, and payment method). Multiple prescriptions for the same patient on the same day in the same facility were considered as one visit in this study. Prescription data were entered using EpiData 3.1. while Stata 16.0 software was employed for data analysis, including descriptive analysis, multivariate analyses, and multilevel logistic regression.

#### Statistical analysis

Quantitative analysis was conducted using Stata 16.0. Descriptive statistics were first used to summarize physician characteristics, perceptions, and attitudes toward diagnostic tools and prescription characteristics. The main outcome in the regression analysis is a binary variable indicating whether a URTIs prescription included at least one antibiotic, defined at the prescription level.

Multilevel logistic regression was used to examine factors associated with antibiotic prescribing for URTIs, accounting for the hierarchical structure of the data (prescriptions nested within physicians, and physicians nested within primary healthcare facilities). Variables were grouped at the patient level (age group, gender), physician level (gender, age, employment status, education, guideline learning, consultation load, consultation duration), and facility level (facility type, CRP-test availability).

Model building was conducted sequentially. First, an empty model with no explanatory variables was fitted to assess the degree of clustering in antibiotic prescribing and to estimate the intraclass correlation coefficient (ICC). Next, physician- and facility-level variables were added, followed by patient-level variables to obtain the fully adjusted model. Odds ratio (OR) and 95% confidence interval (CI) were reported. Model fit was assessed using log-likelihood and deviance statistics. A two-sided p-value of < 0.05 was considered statistically significant.

Variables included in the multilevel models were selected a priori based on the study objectives, the conceptual relevance of diagnostic capacity to antibiotic prescribing, and data availability from the questionnaire and prescription records.

### Qualitative study

Interviews focused on physicians’ demographic characteristics, antibiotic prescribing rationales, perceptions of URTI diagnostic tools, and experiences with antibiotic treatment across different clinical presentations. Interviews were conducted in parallel with prescription audits to directly link prescribing behavior with clinical reasoning. All interviews were conducted in physicians’ offices using a semi-structured guide with open-ended questions, audio-recorded, anonymized, and transcribed within 24 h. A thematic analysis was performed using NVivo 11.0 following three steps: identification of key statements, theme development, and interpretation of thematic relationships. Data reliability was ensured through transcript verification, team-based coding discussions, and maintenance of real-world clinical context throughout the interview process. The interview guide is provided in Additional file 1. C.

## Results

### Quantitative data analysis

#### Socio-demographic characteristics

Most of the GPs from PHC facilities included in the study were female (55.6%). Participants ranged in age from 26 to 68 years, and the largest proportion (32.4%) was aged 40–50 years. Among the participating physicians, 80.6% were permanent employees, and only a small number were contract or part-time staff. Specific socio-demographic characteristics are shown in Table 1.

**Table 1:** Socio-demographic of general practitioners from primary healthcare facilities and characteristics of facilities

Variables		*n*	%	
Physician-level(*n* = 108)				
Gender				
Male	48			44.4
Female	60			55.6
Ages				
≤ 30	14			13.0
31 ~ 40	32			29.6
41 ~ 50	35			32.4
> 50	27			25.0
Educational attainment				
Junior college and below	47			43.5
Bachelor	59			54.6
Master and above	2			1.9
Regular employee				
Yes	87			80.6
No	21			19.4
Guideline learning				
Yes	89			82.4
No	19			17.6
Consultation per day				
≤ 10	41			38.0
11 ~ 30	50			46.2
> 30	17			15.8
Average consultation time				
≤ 5	20			18.5
6 ~ 10	48			44.4
> 10	40			37.1
Physicians by facility type				
Community Health Service Centre	52			48.1
Township Health Service Centre	56			51.9
Institution-level(*n* = 30)				
PHC facilities type				
Community Health Service Centre	16			53.3
Township Health Service Centre	14			46.7
C-reactive protein equipped				
Non-available	19			63.3
Available	11			36.7
Prescription-level(*n* = 12489)				
Prescriptions with antibiotics				
Yes	7706			61.7
No	4783			38.3

#### Perception of diagnostic tools availability

**Fig. 2:**
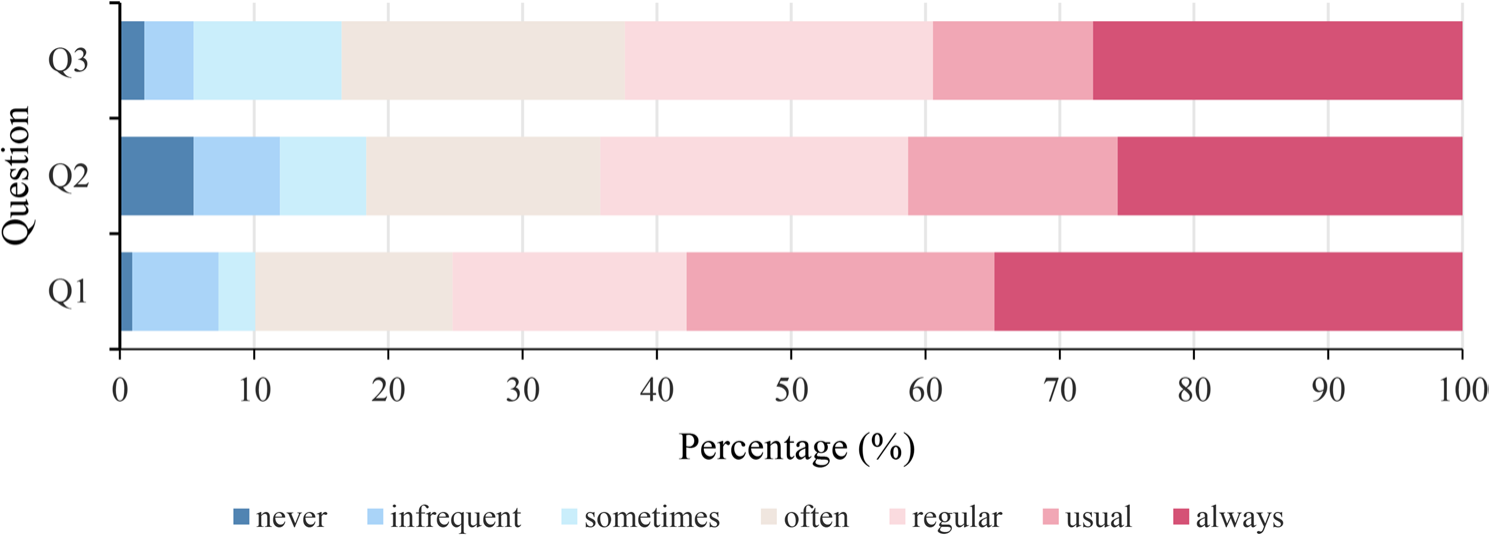
Perception of diagnostic tool availability for URTIs. (Q1: In your experience, how often have you felt that there was a lack of adequate diagnostic testing during the diagnostic process? Q2: Do you think that the lack of diagnostic test results for acute upper respiratory tract infections affects your clinical prescribing decisions? Q3: When formulating a treatment plan, do you feel that more comprehensive diagnostic test results for acute upper respiratory tract infections are necessary?)

Among the 108 respondents, 82.4% (89/108) reported a lack of necessary diagnostic tools for URTIs in routine practice; 79.7% (86/108) reported that lack of diagnostic results at least occasionally influenced their prescribing decisions; and 88.9% (96/108) reported that they at least occasionally needed more complete diagnostic information when formulating treatment plans (Fig. 2).

#### Attitudes towards the use of diagnostic tools

**Fig. 3:**
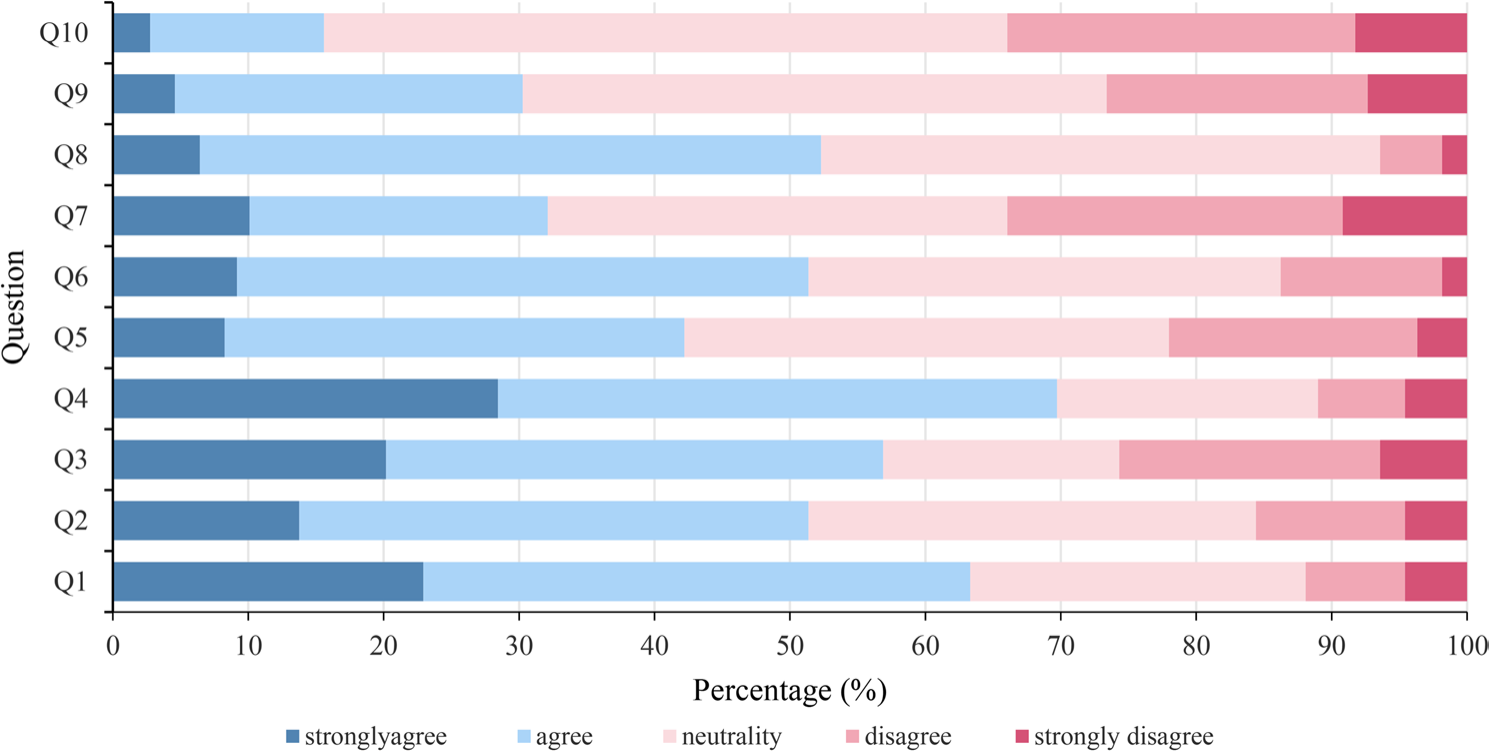
Attitudes towards the use of diagnostic tools for URTIs. (Q1: Ordering diagnostic tests for acute upper respiratory tract infections makes me feel more confident in my clinical decisions; Q2: Ordering relevant diagnostic tests for acute upper respiratory tract infections can improve patient satisfaction; Q3: The results of diagnostic tests for acute upper respiratory tract infections influence my clinical decision-making; Q4: Diagnostic testing for acute upper respiratory tract infections helps shorten the optimal treatment time and reduce related costs; Q5: If I do not order diagnostic tests for acute upper respiratory tract infections, patients may become worried or question my initial diagnosis; Q6: Some patients question the necessity of ordering diagnostic tests for acute upper respiratory tract infections; Q7: It is not difficult for me to appropriately order diagnostic tests for acute upper respiratory tract infections; Q8: The results of diagnostic tests for acute upper respiratory tract infections are generally consistent with my clinical judgment based on experience; Q9: The clinical information obtained from the patient is sufficient for me to make clinical decisions without diagnostic testing; Q10: I have concerns about the reliability of diagnostic test results for acute upper respiratory tract infections.)

Among the 108 respondents, 63.9% agreed that conducting diagnostic tests increased their clinical confidence, 52.8% agreed that ordering relevant diagnostic tests for acute upper respiratory tract infections can improve patient satisfaction, 55.5% agreed that diagnostic results informed their clinical decisions, and 72.2% agreed that diagnostic testing could shorten treatment duration and reduce healthcare costs. By contrast, 41.6% expressed concerns about the necessity of diagnostic testing in some situations, and 14.8% expressed concerns about test reliability. The mean total attitude score was 32.02, indicating an overall moderate attitude towards the clinical use of diagnostic tools (Fig. 3).

Attitude scores toward diagnostic tools ranged from 17 to 42, with an average of 32.02. Overall, 5.6% of respondents demonstrated a positive attitude, 71.3% expressed a neutral attitude, and 23.1% exhibited a negative attitude towards using diagnostic tools in clinical practice.

#### Multilevel mixed-effects logistic regression analysis

Employing multilevel regression model in sequential to full adjustment, several physician, patient, and facility-level factors were independently associated with antibiotic prescribing for URTIs.

At the facility level, institutional characteristics demonstrated the most notable effects according to the main focus of the current study. Availability of CRP was associated with lower odds of antibiotic use (OR = 0.74, 95% CI: 0.56–0.98, *p* = 0.033), suggesting that greater diagnostic capacity may be linked to more rational prescribing (Table 2). Physicians working in Township health service centre were significantly more likely to prescribe antibiotics than those in community health centers (OR = 1.55, 95% CI: 1.21–2.00, *p* = 0.001).

**Table 2:** Multilevel mixed-effects logistic regression analysis of factors associated with antibiotic prescribing for upper respiratory tract infections

variable	OR	95%CI	*P*-value
Physician characteristics			
Gender			
Male	Ref		
Female	0.78	0.59, 1.03	0.085
Age			
≤ 30	Ref		
31 ~ 40	1.03	1.01, 1.33	0.899
41 ~ 50	1.12	0.72, 1.76	0.625
> 50	0.92	0.54, 1.57	0.764
Highest degree			
Junior college and below	Ref		
Bachelor	0.85	0.60, 1.21	0.384
Master	0.21	0.07, 0.57	0.002
Regular employee			
Yes	Ref		
No	0.86	0.62, 1.17	0.328
Guideline learning			
Yes	Ref		
No	0.96	0.68, 1.36	0.804
Consultation per day			
≤ 10	Ref		
11 ~ 30	0.94	0.72, 1.23	0.658
> 30	0.85	0.57, 1.26	0.415
Average consultation time			
≤ 5	Ref		
6 ~ 10	0.79	0.56, 1.11	0.181
> 10	0.72	0.51, 1.02	0.063
Patient characteristics			
Gender			
Male	Ref		
Female	0.93	0.86, 1.00	0.066
Age			
≤ 18	Ref		
19 ~ 30	1.16	1.01, 1.32	0.035
30 ~ 60	1.14	1.03, 1.27	0.011
60 ~ 75	1.02	0.90, 1.16	0.757
Institution characteristics			
Institute type			
Community Health Service Centre	Ref		
Township Health Service Centre	1.55	1.21, 2.00	0.001
C-reactive protein equipped			
Non-available	Ref		
Available	0.74	0.56, 0.98	0.033

At the physicianlevel, we found that holding a master’s degree was strongly associated with lower odds of antibiotic use (OR = 0.21, 95% CI: 0.07–0.57, *p* = 0.002). In our sample, this represented a small subgroup of physicians with higher formal educational attainment than is typically required in primary healthcare settings. Other demographic factors, including gender, age, employment status, guideline learning, consultation load, and consultation duration, were not significantly associated with prescribing behavior (Table 2).

At the patientlevel, adults aged 19–30 years (OR = 1.16, 95% CI: 1.01–1.32, *p* = 0.035) and 30–60 years (OR = 1.14, 95% CI: 1.03–1.27, *p* = 0.011) had higher odds of receiving antibiotics compared with children ≤ 18 years, while gender showed only a borderline association.

The ICC in the unadjusted model indicated that 11.8% of the variance in antibiotic prescribing was attributable to between-physician differences (ICC = 0.118, 95% CI: 0.090–0.154). Sequential adjustment reduced unexplained clustering, with the ICC declining from 0.0992 in Model 1 to 0.0846 in the fully adjusted Model 3, suggesting that included predictors accounted for a meaningful proportion of between-physician variability. Log-likelihood values increased consistently across models (from − 7891.83 in the unadjusted model to − 7522.06 in Model 3), and corresponding deviance values decreased (from 15,783 to 15,044), indicating improved model fit with each stepwise adjustment. (Additional file 1.D)

### Qualitative data analysis

Thematic analysis of the interviews identified four key themes: (1) The influence of diagnostic tools for URTIs on the daily working of GPs; (2) Empirical antibiotic treatment of URTIs; (3) The relationship between diagnostic tests for URTIs and empirical antibiotic treatment; (4) Recommendations for reducing empirical antibiotic therapy.

Semi-structured interviews were conducted in the sampled PHC. A total of 26 physicians were randomly selected from the 108 GPs with antibiotic prescribing authority. Among the interviewees, 57.7% were female. The respondents had an average of 20.7 years of professional experience, with 53.8% having a bachelor’s degree and 92.4% holding junior or intermediate-level professional titles (Table 3).

**Table 3:** Socio-demographic characteristics of the qualitative interview GPs

Category	*n*	%
Gender		
Male	11	42.3
Female	15	57.7
Institute type		
Community Health Service Centre	13	50.0
Township Health Service Centre	13	50.0
Highest degree		
Junior college and below	12	46.2
Bachelor	14	53.8
Work experience (years)		
≤ 10	4	15.4
11 ~ 20	11	42.3
21 ~ 30	9	34.6
> 30	2	7.7
Professional qualifications		
Primary	12	46.2
Medium	12	46.2
Sub-senior	2	7.6

#### Theme one: the influence of diagnostic tools for URTIs on the daily work of GPs

Respondents generally perceived that diagnostic tools have a substantial influence on their daily clinical practice, particularly in supporting clinical decision-making and facilitating communication with patients. Physicians observed that routine blood tests, CRP testing, and other test results are helpful for diagnosis and follow-up treatment. These results also serve as a form of professional protection, enhancing the credibility of treatment decisions when communicating with patients. Physicians perceived that diagnostic findings helped patients better understand their health status, challenged the preconception that antibiotics are necessary for URTIs, and improved overall satisfaction with care.

Respondents believed that when patients have symptoms but can’t determine whether they are infected with bacteria or viruses, diagnostic test results help clarify the etiology and guide appropriate medication decisions. Such results serve as an important clinical basis for determining treatment.

*“Diagnostic tools play an important role in managing URTIs. Many patients present with symptoms that do not necessarily indicate a bacterial infection. When appropriate diagnostic tools are available*,* we can more accurately determine whether the illness is viral. Without such tests*,* misclassification is more likely to occur*,* increasing the risk of inappropriate antibiotic use.”* (Township Health Service Centre, Shang’luo)

*“Overall*,* it does make a difference. For instance*,* when a patient presents with URTI symptoms but the routine blood test results are normal*,* I begin to question my initial decision to prescribe antibiotics. In such cases*,* I would reconsider and conclude that the patient likely has a simple cold rather than a bacterial infection.”* (Township Health Service Centre, Shang’luo)

*“Diagnostic results provide a form of protection for physicians—they serve as clinical evidence. The more detailed the results are*,* the better.”* (Community Health Service Centre, Xi’an)

*“Some patients with upper respiratory tract infections show elevated lymphocyte counts and white blood cell levels*,* which suggest a viral rather than a bacterial infection. In such cases*,* it becomes easier to explain to patients that antibiotics should be avoided whenever possible.”* (Community Health Service Centre, Xi’an)

Respondents indicated that diagnostic test results help facilitate communication with patients by enabling more authoritative explanations and providing objective evidence to challenge the misconception that URTIs require antibiotic treatment. This, in turn, helps strengthen patients’ trust in physicians and reduces unnecessary antibiotic use.

#### Theme two: empirical antibiotic treatment of URTIs

Respondents indicated that relying on experience to prescribe antibiotics is common in primary healthcare facilities, which was confirmed in interviews with GPs in most Township Health Service Centres. Extensive empirical antibiotic treatment is mainly affected by three factors: physician, patient, and system-level factors.

*“Based on the patient’s symptoms*,* mild cases can be treated empirically; if the cough and throat pain are serious*,* I feel antibiotics are necessary to control the inflammation. Using medication directly can also help reduce the financial burden on patients. These are rural residents*,* after all*,* and it’s not easy for them to seek medical care.”* (Township Health Service Centre, Yan’an)

*“For example*,* the biggest obstacle here is that patients are unwilling to undergo any tests. In their view*,* primary healthcare facilities are only for treating minor illnesses. So they don’t understand why tests are necessary for illnesses they consider minor. Patients believe that diagnostic tests are unnecessary expenses in the prescription*,* and often refuse them even when we try to explain the reasoning.”* (Community Health Service Centre, Shang’luo)

*“Our diagnostic facilities do have some limitations. The equipment and testing capacity are relatively limited*,* and we don’t have full-time laboratory staff. Some of the testing methods are outdated*,* and the accuracy isn’t always good enough to meet patients’ expectations.”* (Community Health Service Centre, Shang’luo)

*“The local economy isn’t very well-off*,* so we have to consider patients’ financial situations and prescribe cheaper antibiotics directly.”* (Township Health Service Centre, Yan’an)

A small number of respondents said that relying on experience to prescribe antibiotics may be appropriate in primary healthcare facilities. In situations where diagnostic tools are unavailable or test results are unreliable, empirical treatment can save time and reduce costs, is generally well accepted by patients, and may help prevent potential disputes between physicians and patients.

*“Relying on experience in treatment usually depends on the severity of the patient’s condition and is more appropriate for mild cases. Primary healthcare facilities lack diagnostic equipment*,* so we often have to rely on empirical treatment. If the physicians have extensive work experience*,* they can judge the situation based on the symptoms alone*,* which is more convenient.”* (Township Health Service Centre, Xi’an)

*“When the diagnosis is unclear*,* relying only on experience and the patient’s symptoms and signs can easily lead to misdiagnosis or missed diagnosis. This affects medication decisions and may impact the patient’s subsequent treatment.”* (Community Health Service Centre, Shang’luo)

Some respondents reported that they often struggled to make prescribing decisions. Although they recognized that empirical antibiotic use lacked diagnostic support and could contribute to irrational prescribing, practical constraints frequently forced them to compromise. Others noted that empirical treatment should be applied selectively, depending on the specific clinical situation.

#### Theme three: relationship between diagnostic tests for URTIs and empirical antibiotic treatment

Most respondents reported that in PHC facilities, diagnostic testing for URTIs and relying on experience to prescribe antibiotics are closely interrelated and cannot be considered in isolation. However, they also emphasized that clinical practice sometimes requires flexibility in balancing the two.

*“Yes*,* we can make an initial assessment based on the patient’s symptoms and physical examination*,* and then use diagnostic tests to support our judgment and guide subsequent treatment. However*,* it is impossible to judge the accuracy of the diagnosis at the primary healthcare facilities. Physicians can only evaluate the infection based on their own experience and prescribe medication accordingly*.*”* (Community Health Service Centre, Shang’luo)

*“Auxiliary diagnostic tests and empirical medications have to be considered together*,* and what we do is often based on the patient’s cooperation. If a patient refuses testing*,* only wants medication*,* and expects to recover quickly*,* then we really have no choice.”* (Township Health Service Centre, Yan’an)

#### Theme four: recommendations for reducing empirical antibiotic therapy

Respondents proposed three key recommendations for improving the current situation of empirical antibiotic treatment in primary healthcare facilities: strengthening the training of GPs, popularizing more knowledge on rational antibiotic use among patients, and improving the infrastructure and capacity of primary healthcare facilities.

*“We need to strengthen standardized training for primary healthcare physicians and change how they approach diagnosis and treatment. Only by integrating diagnostic testing with appropriate clinical decision-making can primary care truly improve.”* (Community Health Service Centre, Xi’an)

*“We should help patients understand proper medical and medication knowledge. If diagnostic testing shows no signs of a bacterial infection*,* we need to explain to them that antibiotics are unnecessary for these symptoms.”* (Township Health Service Centre, Yan’an)

*“It would be ideal if laboratory services in primary healthcare facilities were more complete*,* because the role of primary care in outpatient services has been diminishing. With improved diagnostic methods*,* we should make every effort to use medications rationally.”* (Township Health Service Centre, Shang’luo)

### Mixed-methods integration

The qualitative and quantitative findings were integrated across two key dimensions: the impact of limited diagnostic tools for upper respiratory tract infections on the daily work of PHC physicians and the multifactorial determinants of empirical antibiotic prescribing. The results indicated that the absence of appropriate diagnostic tools substantially affects physicians’ routine clinical practice and is closely linked to the continued use of empirical antibiotic treatment for influenza and other URTIs. The details are provided in Table 4.

**Table 4:** Joint display of mixed-methods results

Theme	Quantitative results	Qualitative results	Insights
Provision of URTIs diagnostic tools affect doctors’ daily work?	A total of 82.4% respondent physicians frequently experienced a lack of necessary diagnostic tools for URTIs in clinical practice.	Diagnostic test results are essential to help doctors analyze the type of disease and use medication appropriately. Reduced Patients’ expectations of irrational use of antibiotics.	URTIs diagnostic tools have an impact on the daily work of physicians, helping them to make clinical decisions and to communicate treatment options to patients.
Lack of diagnostic tools and empirical antibiotic treatment for URTIs	79.7% of respondent physicians believed the diagnostic tools shortage impacted clinical decision-making for medication The probability of prescribing antibiotics was lower compared to not being equipped for C-reactive protein testing. (OR: 0.74, *P* < 0.05).	The interaction between diagnostic tests and empirical antibiotic treatment interacts with each other. In the absence of diagnostic tools or inaccurate diagnostic results, the physician will assess the patient’s condition. In cases where there are symptoms suggestive of a bacterial infection, the physician may opt for empirical antibiotic treatment, which can lead to a rapid improvement in the patient’s condition.	The use of antibiotics is affected by the equipment and results of diagnostic tools, but is also affected by the subjective awareness of physicians and patients. Empirical antibiotic treatment is a long-term cumulative problem that needs to be managed from multiple aspects, including personnel, resources, and environment.
Reasons for Empirical Antibiotic Therapy	88.9% of respondent physicians felt comprehensive diagnostic results were essential for effective treatment plans. Compared with those with lower education, doctors with a master’s degree as their highest education were less likely to prescribe antibiotics (OR: 0.21, *P* < 0.05). Young and middle-aged people are more likely to be prescribed antibiotics than those under 18 and over 60 (OR:1.14, *P* < 0.05). Physicians in township health service centres are more likely to prescribe antibiotics than those in cities(OR:1.55, *P* < 0.05)	The cognitive abilities of medical professionals are often limited. In the absence of test results, they may form an erroneous diagnosis and implement an inappropriate treatment plan, such as the direct administration of antibiotics, without fully relying on diagnostic test results. Patients have a distorted perception of PHC facilities and tend to view them as pharmacies. The environment of PHC facilities is inadequate, yet diagnostic equipment and personnel are still lacking. Test results are often inaccurate, and the supply of commonly used drugs is limited, necessitating the use of antibiotics for treatment.	Empirical antibiotic treatment is influenced by physicians, patients, and institutions. It is necessary to simultaneously improve the cognition and attitudes of doctors and patients, and improve the service level of PHC facilities.

## Discussion

The findings of this study suggest that diagnostic availability in primary healthcare is associated with lower antibiotic prescribing for URTIs in PHC facilities in Shaanxi, China. Quantitative results showed that facility-level availability of CRP testing was associated with lower odds of antibiotic prescribing. At the same time, a large proportion of GPs reported inadequate diagnostic support for URTIs and indicated that the lack of diagnostic results influenced their medication-related clinical decision-making. Taken together, these findings suggest that limited diagnostic support may contribute to uncertainty in routine prescribing practice. Same as previous research, indicating that inadequate diagnostic resources increase diagnostic uncertainty and empirical antibiotic prescriptions [25, 26].

However, diagnostic testing should not be interpreted as a standalone solution to inappropriate antibiotic prescribing. Research in antimicrobial stewardship shows that prescribing decisions in primary care are influenced by multiple interacting factors, including diagnostic uncertainty, perceived patient expectations for antibiotics, communication challenges, and organizational context. In this sense, limited diagnostic capacity is likely to be one important contributor rather than the sole cause of inappropriate prescribing [27]. Intervention studies nevertheless suggest that CRP testing can be useful when implemented appropriately. A meta-analysis of primary care studies found that point-of-care CRP testing reduced antibiotic prescribing for respiratory tract infections [28]. In addition, cluster randomized trials have shown that CRP testing, particularly when combined with communication-skills training, can reduce antibiotic prescribing without compromising patient recovery or satisfaction [15]. These studies suggest that CRP testing may help reduce unnecessary antibiotic use, but its impact is likely to depend on how well it is integrated into routine care, including clinician training, communication practices, workflow, and broader stewardship support.

The availability of diagnostic tools not only supports more accurate clinical assessment but also improves communication and trust between physicians and patients. Evidence-based reassurance derived from diagnostic test results helps in mitigating patient expectations for antibiotics, thereby promoting more rational prescribing practices. This finding echoes existing studies that advocate for the integration of diagnostic stewardship as a pivotal component of antibiotic stewardship programs to reduce empirical antibiotic use [29–31]. However, support for increased diagnostic testing was not uniform: only 5.6% of physicians showed a positive attitude overall, and concerns about the necessity of testing were also reported. This suggests that improving diagnostic availability alone may not be sufficient; physicians’ acceptance of testing must also be considered.

The study identifies a prevalent reliance on empirical antibiotic treatment in the absence of diagnostic tools, driven by both physician and patient-related factors. This practice is prevalent in PHC facilities, particularly in resource-constrained settings where diagnostic facilities are limited [32–34]. Respondents reported that empirical treatment was often based on symptomatic assessment and physicians’ clinical experience. While this approach may be necessary in urgent or resource-constrained settings, it may also increase the risk of misdiagnosis and unnecessary antibiotic use, thereby contributing to AMR. The findings therefore suggest that empirical prescribing should, where possible, be guided by clear clinical guidelines and supplemented by available diagnostic information. This dual strategy can optimize patient outcomes while curbing the misuse of antibiotics [35–37].

The integrated findings highlight several practical implications. First, there is an urgent need to improve the availability of diagnostic tools in PHC facilities. The price of CRP testing varies across regions and facility types in China. Based on our preliminary survey of the 30 healthcare facilities included in this study, the average price of CRP testing was 14.45 CNY. Social health insurance reimbursement is determined at the level of a single visit and depends on whether the cumulative cost of this test, combined with other medical services, reaches the local deductible threshold for basic medical insurance. Once the threshold is met, the cost is shared between the insurance scheme and the patient, though the exact reimbursement rate depends on local policies; otherwise, the patient bears the full out-of-pocket expense. This suggests that affordability may not be the only barrier to diagnostic use, and that infrastructure, test availability, and implementation capacity may also play important roles. Strengthening diagnostic infrastructure, such as expanding access to CRP testing, has the potential to substantially reduce inappropriate antibiotic use and enhance clinical decision-making and patient outcomes. Second, training and ongoing professional development for GPs should place greater emphasis on diagnostic stewardship and evidence-based prescribing practices. In addition, our finding is consistent with evidence from previous systematic reviews demonstrating that patient education initiatives in primary care are effective tools for reducing antibiotic prescribing and patient expectations for antibiotics [38, 39]. Third, enhancing public awareness about the risks of AMR and the value of diagnostic testing can promote more rational healthcare-seeking behaviors and improve adherence to medical recommendations. This is also consistent with recent policy research in China highlighting the importance of broader policy support and patient-centred implementation [40].

This study has several strengths. The mixed-methods design enabled a comprehensive assessment of how diagnostic tool availability influences rational antibiotic use. Quantitative data provided a broad picture of prescribing patterns among GPs, while qualitative findings clarified the contextual challenges shaping clinical decision-making. Robust statistical analyses, including univariate and multilevel models, confirmed a significant association between access to diagnostic tools and more rational prescribing. Qualitative interviews further enriched these results by identifying practical barriers and facilitators to diagnostic tool use in PHC facilities.

Nevertheless, several limitations should be acknowledged. First, the cross-sectional design captures data at a single time point, limiting the ability to assess temporal changes in prescribing behaviors or diagnostic tool availability. Prescription records were collected during January–December 2020, a period when China implemented stringent COVID-19 containment measures. These measures led to substantial changes in healthcare-seeking behavior and a marked reduction in outpatient visits. Nationwide data revealed that all-cause health facility visits declined by 63% in February 2020, with only partial recovery observed by June 2020 [41]. Consequently, the antibiotic prescribing patterns observed in this study may not fully reflect routine practice outside the pandemic context. Second, the reliance on self-reported data from GPs could introduce response bias, as participants may over- or underestimate their behaviors and attitudes. Third, the study was conducted in one province of China, namely Shaanxi, which may restrict the generalizability of the findings to other regions with different healthcare infrastructures and clinical practices. Future studies may overcome these limitations by employing longitudinal designs, incorporating objective measures of antibiotic prescribing, and expanding data collection across diverse geographic locations.

## Conclusion

CRP testing and other diagnostic tools may support more rational antibiotic prescribing for URTIs in PHC facilities. The availability of tests may help improve clinical assessment and communication with patients, and alleviate patient reliance on empirical antibiotic prescribing. However, inappropriate antibiotic prescribing is shaped by multiple interacting factors, and improved diagnostic capacity alone is unlikely to be sufficient. Physicians’ attitudes towards diagnostic tools were not uniformly positive, suggesting that wider implementation should also consider clinical acceptance and practical feasibility. Evidence from intervention studies suggests that CRP testing can contribute to reduced antibiotic prescribing when it is integrated with appropriate training, communication, and stewardship support. Strengthening diagnostic infrastructure, supporting GPs through ongoing professional training, and improving public awareness of appropriate antibiotic use may therefore help promote evidence-informed prescribing and support efforts to address AMR.

## Supplementary Information

Supplementary Material 1: A: List of Prescription Extraction Principles. B: Questionnaire for general practitioners. C: Interview Guide for general practitioners. D: Table of Model Fit.

## Data Availability

All data and materials are available from the corresponding author upon reasonable request.

## Abbreviations

AMR: Antimicrobial resistance
PHC: Particularly in primary healthcare
URTIs: Upper respiratory tract infections
CRP: C-reactive protein
GPs: General practitioners
GDP: Gross Domestic Product
ICC: Intraclass correlation coefficient
OR: Odds ratio
CI: Confidence interval
