# Supplementary material for: Role of diagnostic testing in reducing unnecessary antibiotic use for upper respiratory tract infections in Chinese primary healthcare: a mixed-methods study
Source: BMC Prim Care. 2026 May 28;27:284. doi: 10.1186/s12875-026-03398-z (PMC13417845; doi:10.1186/s12875-026-03398-z)
Supplement: Supplementary file 1 — Supplementary Material 1: A: List of Prescription Extraction Principles. B: Questionnaire for general practitioners. C: Interview Guide for general practitioners. D: Table of Model Fit. [file 12875_2026_3398_MOESM1_ESM.docx]

**Role of diagnostic testing in reducing unnecessary antibiotic use for upper respiratory tract infections in Chinese primary healthcare: a mixed-methods study**

**Additional file 1.A List of Prescription Extraction Principles**

| **Content** | **Requirement** |
| --- | --- |
| Prescription extraction time | January 1, 2020 – December 31, 2020 |
| physician | General practitioners who fill out questionnaires properly, have the authority to prescribe antibiotics, and conduct outpatient treatment independently |
| diagnosis | Upper respiratory tract infection, common cold, acute pharyngitis/laryngitis/tonsillitis, acute tracheobronchitis or cough, acute sinusitis, acute otitis media, etc. |
| Sampling | Starting from the 1st of each month, every 2 days one patient's prescription is selected until 15 prescriptions are collected for that month. If there are fewer than 15 prescriptions in that month, all prescriptions for that month are selected. |

**Additional file 1.B Physicians’ Perceptions and Attitudes Toward Diagnostic Tools for Upper Respiratory Tract Infections and Empirical Antibiotic Therapy**

**Part I. Basic Information**

The primary healthcare institution where you work: __________________

1. Your name: __________________

2. Your gender:

A. Male  B. Female

1. Your age:

A. ≤30 years  B. 31–40 years  C. 41–50 years  D. 50–60 years  E. >60 years

1. Your highest educational attainment:

A. Junior college or below  B. Bachelor’s degree  C. Master’s degree or above

1. Your professional title:

A. None  B. Junior  C. Intermediate  D. Senior

1. Years of medical practice:

A. ≤10 years  B. 11–20 years  C. 21–30 years  D. >30 years

1. Are you a formally employed (established) staff member of the institution?

A. Yes  B. No

1. Have you studied the Primary Care Guidelines for the Diagnosis and Treatment of Acute Upper Respiratory Tract Infections?

A. Yes  B. No

1. Does your institution have on-site laboratory testing services?

A. Yes  B. No

1. Is your institution equipped with C-reactive protein (CRP) testing?
2. Yes  B. No
3. Your average number of patient visits per day:

A. ≤10  B. 11–20  C. 21–30  D. 30–50  E. >50

1. Your average consultation time per patient:

A. ≤5 minutes  B. 6–10 minutes  C. 11–20 minutes  D. >20 minutes

**Part II. Perceptions of the Lack of Diagnostic Tools for Upper Respiratory Tract Infections**

Note: Diagnostic tools for acute upper respiratory tract infections include peripheral blood routine tests, C-reactive protein (CRP), and procalcitonin (PCT), among others.

The following statements describe physicians’ perceptions regarding the lack of diagnostic tools. Please indicate the extent to which you agree with each statement based on your actual experience.

Response options:

1 = Never  2 = Rarely  3 = Sometimes  4 = Occasionally  5 = Often  6 = Usually  7 = Always

1. In your experience, how often have you felt that there was a lack of adequate diagnostic testing during the diagnostic process?

1 ☐ 2 ☐ 3 ☐ 4 ☐ 5 ☐ 6 ☐ 7 ☐

1. Do you think that the lack of diagnostic test results for acute upper respiratory tract infections affects your clinical prescribing decisions?

1 ☐ 2 ☐ 3 ☐ 4 ☐ 5 ☐ 6 ☐ 7 ☐

1. When formulating a treatment plan, do you feel that more comprehensive diagnostic test results for acute upper respiratory tract infections are necessary?

1 ☐ 2 ☐ 3 ☐ 4 ☐ 5 ☐ 6 ☐ 7 ☐

**Part III. Attitudes Toward the Use of Diagnostic Tools for Acute Upper Respiratory Tract Infections**

Note: The main presenting symptoms of patients with acute upper respiratory tract infections include fever, headache, sore throat, cough, and other respiratory manifestations.

The following statements describe physicians’ attitudes toward the use of diagnostic tools for acute upper respiratory tract infections. Please indicate your level of agreement with each statement based on your actual experience.

Response options:

Q1-5(positively worded and scored directly):1 = Strongly disagree  2 = Disagree  3 = Neutral  4 = Agree  5 = Strongly agree

Q6-10(negatively worded and reverse-scored):1 = Strongly agree  2 = Agree  3 = Neutral  4 = Disagree  5 = Strongly disagree

1. Ordering diagnostic tests for acute upper respiratory tract infections makes me feel more confident in my clinical decisions.

1 ☐ 2 ☐ 3 ☐ 4 ☐ 5 ☐

1. Ordering relevant diagnostic tests for acute upper respiratory tract infections can improve patient satisfaction.

1 ☐ 2 ☐ 3 ☐ 4 ☐ 5 ☐

1. The results of diagnostic tests for acute upper respiratory tract infections influence my clinical decision-making.

1 ☐ 2 ☐ 3 ☐ 4 ☐ 5 ☐

1. Diagnostic testing for acute upper respiratory tract infections helps shorten the optimal treatment time and reduce related costs.

1 ☐ 2 ☐ 3 ☐ 4 ☐ 5 ☐

1. If I do not order diagnostic tests for acute upper respiratory tract infections, patients may become worried or question my initial diagnosis.

1 ☐ 2 ☐ 3 ☐ 4 ☐ 5 ☐

1. Some patients question the necessity of ordering diagnostic tests for acute upper respiratory tract infections.

1 ☐ 2 ☐ 3 ☐ 4 ☐ 5 ☐

1. It is not difficult for me to appropriately order diagnostic tests for acute upper respiratory tract infections.

1 ☐ 2 ☐ 3 ☐ 4 ☐ 5 ☐

1. The results of diagnostic tests for acute upper respiratory tract infections are generally consistent with my clinical judgment based on experience.

1 ☐ 2 ☐ 3 ☐ 4 ☐ 5 ☐

1. The clinical information obtained from the patient is sufficient for me to make clinical decisions without diagnostic testing.

1 ☐ 2 ☐ 3 ☐ 4 ☐ 5 ☐

1. I have concerns about the reliability of diagnostic test results for acute upper respiratory tract infections.

1 ☐ 2 ☐ 3 ☐ 4 ☐ 5 ☐

**Part IV. Attitudes Toward Empirical Antibiotic Therapy for Acute Upper Respiratory Tract Infections**

Note: Empirical antibiotic therapy refers to the practice whereby physicians rely on patients’ self-reported symptoms of suspected acute upper respiratory tract infections and their own prior clinical experience to infer the pathogenic cause, without using relevant auxiliary diagnostic tests, and directly initiate treatment with antibiotics.

The following statements describe physicians’ attitudes toward empirical antibiotic therapy. Please indicate your level of agreement with each statement based on your actual experience.

Response options:

1 = Strongly agree  2 = Agree  3 = Neutral  4 = Disagree  5 = Strongly disagree

1. Empirical antibiotic therapy for acute upper respiratory tract infections is necessary in clinical practice.

1 ☐ 2 ☐ 3 ☐ 4 ☐ 5 ☐

1. Based on patients’ clinical symptoms and physical examination findings, empirical antibiotic therapy for acute upper respiratory tract infections can be appropriately initiated.

1 ☐ 2 ☐ 3 ☐ 4 ☐ 5 ☐

1. Even when the reliability of diagnostic test results is low or when diagnostic technologies are limited, empirical antibiotic therapy for acute upper respiratory tract infections is still necessary.

1 ☐ 2 ☐ 3 ☐ 4 ☐ 5 ☐

1. Patients with acute upper respiratory tract infections prefer to receive empirical antibiotic therapy based on symptoms, as it helps them recover faster while saving time and money.

1 ☐ 2 ☐ 3 ☐ 4 ☐ 5 ☐

1. Empirical antibiotic therapy for acute upper respiratory tract infections is more suitable for routine practice in primary healthcare institutions.

1 ☐ 2 ☐ 3 ☐ 4 ☐ 5 ☐

1. Empirical antibiotic therapy for acute upper respiratory tract infections is simple and efficient and helps improve work efficiency.

1 ☐ 2 ☐ 3 ☐ 4 ☐ 5 ☐

1. The safety of empirical antibiotic therapy for acute upper respiratory tract infections can be adequately ensured.

1 ☐ 2 ☐ 3 ☐ 4 ☐ 5 ☐

1. In most cases, my colleagues and I tend to choose empirical antibiotic therapy for acute upper respiratory tract infections.

1 ☐ 2 ☐ 3 ☐ 4 ☐ 5 ☐

1. Empirical antibiotic therapy for acute upper respiratory tract infections generally involves the use of broad-spectrum antibiotics to alleviate symptoms.

1 ☐ 2 ☐ 3 ☐ 4 ☐ 5 ☐

1. When the symptoms are atypical, I usually prescribe medication based on empirical judgment first and conduct further diagnostic testing only if the patient does not improve.

1 ☐ 2 ☐ 3 ☐ 4 ☐ 5 ☐

1. Empirical antibiotic therapy for acute upper respiratory tract infections increases patients’ medication burden (including unnecessary treatment).

1 ☐ 2 ☐ 3 ☐ 4 ☐ 5 ☐

1. Relying solely on clinical experience may lead to misdiagnosis or missed diagnosis.

1 ☐ 2 ☐ 3 ☐ 4 ☐ 5 ☐

**Additional file 1.C Interview Guide on Physicians’ Perceptions and Attitudes Toward Diagnostic Tools for Upper Respiratory Tract Infections and Empirical Antibiotic Therapy**

**Part I. Basic Information**

The primary healthcare institution where you work: __________________

1. Your gender:

A. Male  B. Female

1. Years of medical practice:

A. ≤10 years  B. 11–20 years  C. 21–30 years  D. >30 years

1. Your highest educational attainment:

A. Junior college or below  B. Bachelor’s degree  C. Master’s degree or above

1. Your professional title:

A. None  B. Junior  C. Intermediate  D. Senior

**Part II. Decision-Making Basis for Prescribing Antibiotics**

1. What is your usual clinical workflow when diagnosing and treating patients with upper respiratory tract infections in daily practice?
2. How do you determine the type of infection in your patients?
3. In your opinion, what factors form the basis for your decision to prescribe antibiotics?

**Part III. Views on Diagnostic Tools for Upper Respiratory Tract Infections**

1. During the consultation process, do you think it is necessary to order auxiliary diagnostic tests? Under what circumstances do you think auxiliary tests should be ordered, and under what circumstances are they unnecessary?
2. How do you evaluate the reliability of results from tests such as routine blood tests and C-reactive protein (CRP)?
3. What impact do you think auxiliary diagnostic tools have on your daily clinical practice?

**Part IV. Views on Empirical Antibiotic Therapy**

1. What is your overall attitude toward empirical antibiotic therapy?
2. In your opinion, what are the main reasons for the use of empirical antibiotic therapy in primary healthcare institutions?
3. How do you perceive the relationship between “auxiliary diagnostic tools” and “empirical antibiotic therapy”?
4. What suggestions or recommendations do you have for reducing irrational empirical antibiotic use in primary healthcare institutions?

**Additional file 1.D Random Effects and Model Fit in Multilevel Analysis of Antibiotic Use**

| **Parameter** | **Unadjusted** | **Model 1** | **Model 2** | **Model 3** |
| --- | --- | --- | --- | --- |
| Log-likelihood | -7891.8277 | -7530.1929 | -7878.4958 | -7522.0576 |
| ICC | 0.118  (0.0904, 0.154) | 0.0992  (0.0742,0.131) | 0.0925  (0.0695,0.122) | 0.0846  (0.0627,0.113) |
| Deviance | 15783 | 15060 | 15757 | 15044 |
